# Supplementary material for: p38 Mitogen-Activated Protein Kinase Inhibition of Mesenchymal Transdifferentiated Tumor Cells in Head and Neck Squamous Cell Carcinoma
Source: Biomedicines. 2023 Dec 13;11(12):3301. doi: 10.3390/biomedicines11123301 (PMC10741606; doi:10.3390/biomedicines11123301)
Supplement: Supplementary file 1 [file biomedicines-11-03301-s001.zip › biomedicines-2714000-supplementary.pdf]

Supplementary Figure S1: SB202190 and TGF-beta-1 effects on normalized p-ATF2 phosphoprotein levels in SCC-25 cells.

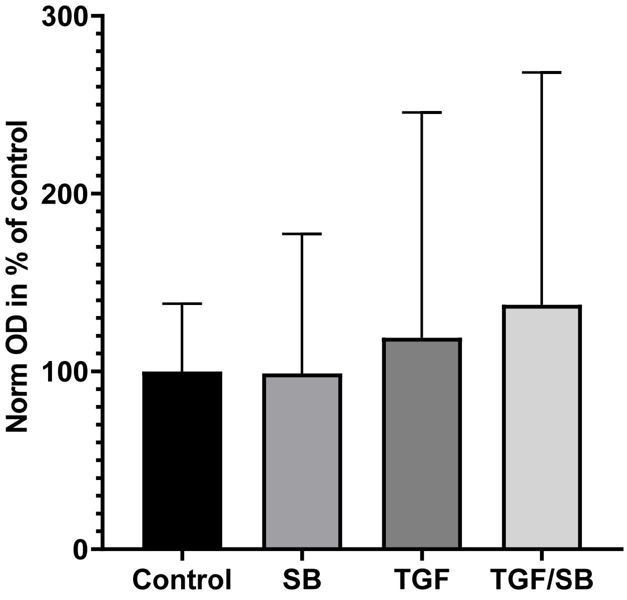

Figure S1. SCC-25 cells were treated with DMEM/F12 control medium (1), SB202190 (2), TGF-beta-1 (3) and TGF-beta-1 plus SB202190 (4). p-ATF2 protein was analyzed by Western blot in 10 independent biological repeats. Immunoblotting reactions were visualized by highly sensitive horseradish peroxidase (HRP)-labeled secondary antibodies and chemiluminescent substrate reaction. GAPDH was used as loading control and detected with NIR fluorescence. Phospho-ATF2 optical densities were normalized with those of GAPDH. The normalized optical densities (OD) in all experiments were related to mean of the controls, which was considered 100%. The relative optical densities of control and treatments (n=10 in all cases) are presented on a column chart. No significant differences were found among the control and treatment datasets (p=0.84 by Kruskal-Wallis test).
